# Student feedback about the use of role plays in Sparshanam, a medical humanities module

**DOI:** 10.12688/f1000research.1-65.v1

**Published:** 2012-12-13

**Authors:** P Ravi Shankar, Rano M Piryani, Kundan K Singh, Bal Man S Karki

**Affiliations:** 1 Department of Medical Education, KIST Medical College, Lalitpur, Nepal; 2 Department of Medicine, KIST Medical College, Lalitpur, Nepal; 3 Department of Clinical Pharmacology, KIST Medical College, Lalitpur, Nepal; 4 Director of Academics, KIST Medical College, Lalitpur, Nepal

## Abstract

**Background:** At KIST Medical College, Lalitpur, Nepal, a Medical Humanities module for first year medical students has been conducted. Role plays are used to explore social, medical and sexual issues in the Nepalese context. The present study obtained student feedback about the role plays used in the module, the difficulties faced, and obtained suggestions for further improvement.

**Method: **The module was conducted from January to August 2011 using a total of 15 role plays. Student feedback was obtained using a semi-structured questionnaire. Informal discussions were held and a questionnaire was circulated among the first year students who had participated in the module.

**Results:** Ninety-eight of the 100 students in the module participated in the study. The overall opinion regarding the role plays was positive. Students stated role plays helped to make module objectives concrete and interesting, made students identify with the problem being investigated and improved communication skills. Role plays were designed to address important health issues in Nepal and prepare students for addressing these issues in future practice. A lack of sufficient time for preparing the role plays and initial problems with group dynamics were mentioned by the respondents during the study.

**Conclusions:** Student feedback about the use of role plays during the module was positive. Role plays helped in making module objectives more concrete and interesting, improved communication skills and addressed important health issues in Nepal. Role plays are not resource intensive and can be considered for use in medical schools in developing nations.

## Background

Medical humanities (MH) use subjects traditionally known as the humanities to explore issues in medical education. Literature, painting, sculpture, music, anthropology, philosophy and related subjects are used in MH modules throughout the world. MH supports the exploration of the human side of medicine and is the intersection of the arts and medicine ^1^. MH programs are uncommon in South Asia. A voluntary MH module was conducted at Manipal College of Medical College (MCOMS), Pokhara, Nepal ^2^. For the past three years we have also been conducting a MH module at KIST Medical College (KISTMC), Lalitpur, Nepal, first for faculty and medical/dental officers and later for first year medical students ^3^. The module uses small group, activity-based sessions to explore various aspects of the humanities. Case scenarios, role plays, debates and interpretation of paintings are among the different learning modalities used.

Role plays have been shown to promote active learning of students and are an experiential learning technique with learners acting out roles in case scenarios to provide targeted practice and feedback to train skills ^4^. In Nepal, the use of role plays in educating medical students is limited. However they have been used in certain institutions. At MCOMS, Pokhara role plays were/are used to teach students to communicate non-drug and drug measures with respect to common conditions/diseases using simulated patients by the department of Pharmacology ^5^. At KISTMC, the department of Clinical Pharmacology uses role plays for the same purpose and to optimize the time spent with medical representatives ^6^. At MCOMS, role plays were used to explore issues of human sexuality during the voluntary MH module and during a session on social issues in the use of medicines ^7^. In New Delhi, Indian medical students had used drama to explore the emotional pressures on the humane dimension of a medical student’s life ^8^.

When role plays are used in an unplanned and ad hoc manner, students often report dissatisfaction as active learning is impaired ^4^. However, role plays have many potential uses in medical education. Of special relevance to MH are role plays enabling students to place themselves in situations they have not experienced before, to help them empathize and understand other people’s problems and motivations ^9^.

Role plays have been used to teach students the skill of breaking bad news ^10^. The most effective educational interventions present basic steps to delivering bad news and provide opportunities to learners to discuss their concerns, to enable them to practice, and to receive feedback on their skills ^11^. In Germany, video-taped role plays and subsequent analyses are used to teach students the skill of breaking bad news ^12^. At the University of Heidelberg, Germany, introducing role plays enhanced the realism of technical training and improved doctor-patient communication ^13^. In a Malaysian medical school, role plays have been used to teach communication skills in primary care medicine ^14^ and to teach students to obtain a sexual history and discuss sexual health issues ^15^. At MCOMS role plays were used to teach students to critically evaluate drug promotion ^16^.

In Nepal, students enter medical school after twelve years of schooling with the subjects of physics, chemistry, biology and English being compulsory during the last two years. Students do not have much exposure to life situations and are emotionally immature. In our institution, early clinical exposure for four hours every week starts right from day one. The scenarios used in the MH module and their interpretation by students using role plays exposed them to situations that they are unlikely to have encountered in their life. MH introduces students to problematic life situations, teaches students to communicate better, and can stimulate creativity and the imagination ^17^. Role plays enable students to place themselves in the situation of another person and may help to develop empathy. Role plays early in the course can expose students to different situations they are likely to face in their future career. Students become aware of social issues and other problems of the country so that they can be more active citizens. In Nepal, other medical schools are slowly adopting role plays for specific purposes in medical education. The Patan Academy of Health Sciences (PAHS) has the mission to train doctors for rural Nepal and community health sciences are an important part of the curriculum ^18^. The institution encourages reflection and is planning to introduce MH in its curriculum.

The module for the third batch of students concentrated on five important areas. The first session was on empathy and the second one on ‘What it means to be sick in Nepal’. The other sessions were on the doctor, the patient and the doctor-patient relationship. There was also a concluding session. The module was held on alternate thursdays for around two hours for a group of 50 students. The students were divided into five small groups and facilitator presentations, case scenarios, role plays, paintings, and student presentations and activities (such as identifying learning issues from case scenarios and interpreting them using role plays, interpreting paintings and photographs among others) were among the different teaching-learning modalities used. Student feedback on the paintings used during the module in 2012 has been published ^19^. Use of more paintings from Nepal and South Asia was suggested by the respondents in the study ^19^. Following this study, we are now using paintings by our medical students which were exhibited during an art exhibition in the institution.

The content of role plays was often designed around social and political situations in contemporary Nepal. Nepal is recovering from a decade-long conflict and the scars are still visible. Many parts of the country were mined by the two sides and while the problem is not as extensive as in many other nations, land mines can be a danger to innocent civilians. Trafficking of young girls continues to be a problem though many organizations are now active in preventing trafficking and rescuing young girls ^20^. Leprosy and HIV/AIDS continue to be diseases with significant social stigma and the status of women continues to be poor.

In the United States of America (USA), ‘The art of doctoring’, was introduced as an elective module to third and fourth year students ^21^. Role plays were used along with other learning modalities. Role plays have also been used to teach appropriate interaction with pharmaceutical company representatives ^22^.

Detailed participant feedback on the role plays used in the MH module had not previously been obtained. Hence the present study was carried out with the following objectives:

a) To obtain participant feedback about role plays used in Sparshanam.

b) To understand problems and difficulties in interpreting and enacting the role plays.

c) To obtain suggestions for further improving the use of role plays during future modules.

## Methods

The topics covered during the MH module were empathy, what it means to be sick in Nepal, the doctor, the patient and the doctor-patient relationship. Each topic was completed in two sessions called ‘bytes’. The module and the role plays were conducted using resources available in the institution. The role plays were video recorded with the consent of the participants.

The third intake of undergraduate medical (MBBS) students joined the course in November-December 2010 and Sparshanam, the MH module, was conducted from January to early August 2011. The intake of 100 students was subdivided into two batches and sessions were held for each batch on alternate thursdays. The large groups of 50 students were then divided into five small groups of 10 students each.

The case scenarios were provided to the small groups, who analysed the scenario, identified the learning issues and tried to interpret the various issues using a role play. Students were given 10 minutes for preparation but they often needed more time (up to 15 minutes) which was provided. A group member introduced the role play and the actors. Participants were debriefed regarding their feelings and emotions while playing a particular role after certain role plays. We used 15 role plays during the module. Table 1 shows the scenarios used during the module. These scenarios were agreed upon by the authors to address common and important problems in the country.

**Table 1.** Role plays used during the medical humanities module.

Name of session	Role plays used
What it means to be sick in Nepal (byte 1)	Ms. Anita is a 35 year old lady living in Bungamati, Lalitpur. She had contracted poliomyelitis as a child. Her legs are withered and she is unable to walk. She makes her living by begging for alms in public places. Explore what it means to be sick in Nepal using a role-play. Mr. Abhiram is a poor farmer living in Dhanusha district. His two year old son has been suffering from diarrhea over the last two days. The family is uneducated and believes that if water is given during diarrhea it would be lost in the stools. The boy, Suraj has not been given anything by mouth over the last two days. He is extremely weak, stupurous and dehydrated. Explore what it means to be sick in Nepal using a role-play. Ms. Mohini is a 28 year old lady who was trafficked to India and was compelled to become a commercial sex worker. After ten years of service she was sent back to her village as she became HIV positive. The disease is at an advanced stage and she has no money for treatment. Her family has reluctantly allowed her to stay with them but is not happy that a retired prostitute is living with them. Explore what it means to be sick in Nepal using a role-play.
What it means to be sick in Nepal (byte 2)	James & Anil are a gay couple living in Pokhara. They work in a travel agency in Lakeside. Recently they have been diagnosed to be HIV positive. Their coworkers are scared and want the couple to be dismissed from their jobs. Highlight the issues involved. Explore the issues involved using a role play. Julie is the daughter of rich parents. She is ashamed of her Mongolian features and wants to undergo plastic surgery to get classical Aryan features. Her boyfriend also is in favor of this. Highlight the issues involved. Explore the issues involved using a role play. Sukh Maya and Bir are a couple living in a remote village in mid-western Nepal. Their fourteen year old daughter is suffering from high fever and convulsions. The nearest health facility is over a day’s walk away. The couple believe their daughter has been possessed by an evil spirit and are reluctant to seek medical treatment. Highlight the issues involved. Explore the issues involved using a role play.
The patient (byte 1)	Ram is a 30 year old man who has been diagnosed to be suffering from leprosy. He lives in a small village in eastern Nepal. On learning that he is suffering from leprosy his wife has left him taking with her their children. The villagers shun Ram and his family. He is very depressed and has come to you, the doctor in a PHC an hour’s walk from the village accompanied by his mother. The PHC staffs are not very happy that a leprosy patient has come to their hospital. Explore the scenario from the patient perspective using a role-play. Anita is a married lady living in a remote village in Baglung district. While going out to collect fodder for her cattle she stepped on a land mine, a relic of the decade long violent conflict. Her right leg was badly damaged. She is admitted to your hospital and you are the treating doctor. You have to amputate her limb from above the knee to save her life and gangrene and infection have set in the limb. Explore the scenario from the patient perspective using a role-play. Asmita is a young lady who has just delivered a baby in Achham district of far-Western Nepal. The family considers her unclean and following the age old ‘Chaupadi’ system has banished her and the newborn to the family cow shed. Asmita is extremely depressed and malnourished. Explore the scenario from the patient perspective using a role-play.
The doctor (byte 1)	Dr. Ram Bahadur is a famous doctor in Pokhara. He has a busy practice and runs a flourishing nursing home. He is busy from morning till night in his practice. His wife is extremely unhappy. She accuses him of having no time for her and the family. He is also accused of being money minded. She wants to divorce him. Explore the situation using a role-play. Dr. Bimalendra is a famous gynecologist in Lalitpur. He has a flourishing practice. Recently he was accused of behaving improperly with a female patient. The patient’s relatives are very angry and agitated and have come to the hospital with the intention of manhandling the doctor and teaching him a lesson. Explore the situation using a role-play. Dr. Anita is a doctor with a roaring practice. She has been approached by a pharmaceutical company with a loyalty scheme. On prescribing the company’s products the doctor earns points and depending on the points the rewards can range from a refrigerator, a car to an all-expenses paid trip to Las Vegas with a companion. The company’s representative is in the doctor’s office to convince her to join the scheme. Explore the situation using a role-play.
Doctor-patient relationship (byte 1)	Dr. Kiran is an Internal medicine specialist in Lalitpur. He has been treating a twenty-two year old college student named Anita for the last five years. The lady suffers from severe attacks of migraine and is on drug prophylaxis. Kiran has realized that he is in love with Anita. He wants to live happily ever after with her. However, he is not sure about whether it would be correct for a doctor to marry his young female patient. Analyze the issues involved using a role play. Irena is a young lady suffering from the terminal stage of cancer. She was/is in a lesbian relationship with an older lady. She is in severe pain and her once beautiful body has been reduced to a skeleton. Her lover and family cannot bear to see Irena in pain and want you, their family physician to put Irena out of her misery. Analyze the issues involved using a role play. Dr. Chitrakar is a Psychiatrist practicing in Lalitpur. A social worker had contacted him recently with the story of a young woman who is believed to be a ‘mental case’ and has been locked away in the top floor of her family’s house in Lubhu. The social worker is very concerned about the young woman and has exerted considerable pressure on the family to visit you in your clinic. You have decided to stress that mental disease is an illness like any other and can be treated in your discussion with the family. Analyze the issues involved using a role play.

The scenarios and the transcripts of certain role plays mentioned has been recently published in the International Journal of User-driven Healthcare ^23^.

Students worked in small groups. These groups were kept constant throughout the module. As facilitators, we gave students freedom to explore the role plays according to group opinion and consensus and did not impose our opinions on the group. The scenarios were distributed to the group who then discussed which issues to explore and how to present the role play. Facilitators were present in the background to offer support when requested.

This study was conducted at the end of July and early August 2011. A semi-structured questionnaire was administered to students. Written informed consent was obtained from all participants. The study was approved by the Institutional Review Board (IRB). The questionnaire was tested for readability and ease of understanding among four faculty members and four second year students. The questionnaire used in the study is shown below.

Questionnaire student feedback about the use of role plays Questionnaire used to determine student enjoyment and usefulness scores of role plays used during the medical humanities at KIST Medical College, Nepal

The gender and method of financing of medical education was noted. Self-financing students have to pay high tuition fees and tend to be from a higher socioeconomic group compared to scholarship students. Ninety students are self-financing and 10 seats are offered on a full tuition fee scholarship to candidates selected by the Ministry of Education through an entrance exam. Overall comments about use of role plays in Sparshanam, whether students had been exposed to role plays before, and how role plays helped in realizing the objectives of the module were studied. A brief description of all role plays conducted during the module was shown to students on power point slides while filling the questionnaire.

The participant responses were collected and grouped together and the number of respondents stating each response was noted. Responses were either quoted verbatim by the authors or paraphrased in the findings. The responses quoted verbatim have been put in single inverted commas in the results. For the two ratings the mean score was calculated and compared among subgroups using independent samples t-test. A p value <0.05 was taken as statistically significant.

## Results

Ninety-eight of the 100 students (98%) participated in the study; 53 (54.1%) were male and 43 (43.9%) were female. Two respondents did not mention their gender. Eight students (8.2%) were scholarship students while 90 were self-financing.

Table 1 shows the role plays used during the medical humanities module. Table 2 shows the paraphrased common overall comments about the module. Respondents felt the module was successful in providing knowledge about health and social issues in Nepal. Twenty-seven students (27.5%) were exposed to role plays for educational objectives in school. Seven of these 27 students had done role plays to raise public awareness: among the issues they had covered were drug abuse, education of girls, and trafficking of women. Sixty-eight students had not been exposed to role plays before.

**Table 2.** Common overall comments about the medical humanities module.

**Comment**	**Number of** **respondents**	**Percentage of** **total (n = 98)**
Gave knowledge about health and social issues in Nepal	26	26.5
Was a refreshing break from the routine	25	25.5
Was interesting	25	25.5
Taught how to deal with future situations	17	17.3
Provided familiarization with problems in professional life	13	13.3
Was interactive	9	9.2
Developed team spirit among team members	8	8.2
Helped in clarifying session objectives	7	7.1

According to respondents, role plays helped in realizing the module objectives in many ways. Among the strengths of role plays mentioned were ‘live interaction in real situations’ [n = 27 (27.5%)], ‘made objectives more interesting’ [n = 16 (16.3%)], ‘we understood different aspects of the problem’ [n = 9 (9.2%)] and ‘sessions improved verbal and non-verbal communication’ (n = 6). Fifteen respondents (15.3%) were aware of the use of role plays elsewhere while 72 were not aware of this. In Nepal, the medical schools mentioned as having role plays by the students as a teaching-learning methodology were Patan Academy of Health Sciences (PAHS) (n = 5), and one each mentioned Manipal College of Medical Sciences, Pokhara, and Nepal Medical College. Fifty-two respondents (53.1%) rated their enjoyment of role plays in the module as 4/5 while 39 (39.8%) rated it as 5/5. The mean ± SD enjoyment score was 4.26 ± 0.94. There was no significant difference in the mean score according to gender or method of financing of medical education.

Eighty-seven respondents (88.8%) felt the use of role plays was appropriate. Among the reasons provided were role plays covered different health and social issues in the country (n = 39), the issues covered will be experienced in future practice (n = 33), and they dealt with issues of importance to the doctor-patient relationship in Nepal (n = 6). Table 3 shows the paraphrased suggestions of respondents to make role plays more useful. Table 4 mentions the role plays with which respondents identified the most.

**Table 3.** Suggestions of respondents to further improve use of role plays in future.

**Suggestion**	**Number of** **respondents**	**Percentage**
Give more time for preparation and planning of the role plays to the groups	55	56.1
Provide different scenarios to different groups	19	19.4
Resolve technical problems like microphones and seating	18	18.4
Create and provide more scenarios	16	16.3
Ensure more participation of students	14	14.3
Create more characters in the role play so that more students can be involved	13	13.3
Use more role plays in the medical humanities module	10	10.2
Create more realistic scenarios	8	8.2
Inform the students about the scenarios at least a day in advance	7	7.1

**Table 4.** Role plays with which respondents identified the most.

**Role play**	**Number of** **respondents (%)**	**Reason**
Lady stepping on land mine and lower limb was amputated	18 (18.4)	Well presented, good mimicry of sound of ambulance, legacy of conflict
Trafficking of young girl to India for sale to a brothel	16 (16.3)	Major problem in Nepal, good acting, sister is a volunteer in NGO addressing this problem
Leprosy patient whose wife has left him	15 (15.3)	Major problem in Nepal, Social stigma
Gay couple who are HIV-positive and co-workers want them removed from their job	13 (13.3)	Brought out problem of gays, was a part of the role play
Chaupadi system where women are banished to the cow shed during menstruation and just after delivery	12 (12.2)	Big problem in certain areas, usually ignored
Doctor falling in love with a patient being treated for many years for migraine	10 (10.2)	Clear objectives and role play was humorous

Student enjoyment and usefulness scores of role plays in a Medical Humanities module at KIST Medical College, Lalitpur, Nepal Enjoyment and usefulness scores of role plays used during the medical humanities module (KIST Medical College, Nepal) according to gender and method of financing of medical education.Gender: 1 = male, 2 = femaleMethod of financing: 1 = self-financing, 2 = scholarship
 Enjoyment and usefulness scores are scored between 1 (lowest) to 5 (highest).

Participants had problems in identifying with the problem of the gay couple (n = 20). Among the reasons cited were that participants felt the problem is less important because it is less prevalent in the country and is not commonly discussed in society. This role play was interpreted differently by different persons with a substantial number also identifying with it. The other role plays that students found difficulty in identifying with were surgery for Aryan features, the problem of leprosy and of mental illness.

Among the difficulties which students had while planning and enacting the role plays were lack of time (n = 18), problems in working together as a team during the initial days when participants did not know each other well (n = 6), occasionally uncooperative group members (n = 6) and problems in deciding who will act in the role plays (n = 5). These were overcome by group discussion and arriving at a consensus in the group (n = 15), dividing work among group members (n = 9), using a lottery system to select actors (n = 7) and respondents who were shy transformed themselves with the help of others (n = 5). Most small groups were able to resolve their initial problems and work together as a team. During each session, each team democratically elected a team leader, a time keeper, a scribe and a presenter. These roles were rotated during subsequent sessions. There were occasional difficulties in playing roles and students were sometimes reluctant to play roles with a sexual and reproductive component. The lottery system was followed by many groups to select actors and was accepted as fair. Despite a certain degree of reluctance and apprehension in playing and tackling sexual issues, respondents agreed these should be addressed during the MH module.

Ninety-two respondents (94%) felt sexual and reproductive issues should be addressed in learning sessions using role plays. Among reasons provided were as doctors they will face these problems in future practice (n = 14), these issues are an important and integral part of medicine (n = 11), and these ‘hidden’ issues are responsible for many problems (n = 8). Fifty respondents opined that these issues sometimes created problems in enacting the role plays compared with 41 respondents who felt that no problems were created. Among the problems mentioned were in acting, people felt awkward due to their conservative nature, and it was hard to create dialogs. These problems were overcome by seeking help from and involving all group members, overcoming initial apprehension in communicating with the opposite gender, using the lottery system to select actors and looking at the issues in a professional manner.

Ninety-six respondents (98%) wanted to undertake role plays in future activities. Among the stated advantages were that students will find it easier to deal with patients in future (n = 29), role plays helped in realizing module objectives (n = 22), students were familiarized with health and social problems of Nepal (n = 19), role plays were a refreshing break from routine (n = 18), role plays will improve communication skills (n = 18), and issues in the doctor-patient relationship were introduced (n = 14).

No significant difference in scores according to demographic characteristics was noted. Among free text comments about the module, one respondent stated: ‘It is appreciable and needs to be continued with more and more innovative ideas. Many other persons from other departments can be included as facilitators’. Another stated: ‘It was a great fun in medical humanities session. I had never had such interesting learning sessions before. It was much more effective and I hope to have such sessions in future also’. Other comments were: ‘Special thanks to the facilitators for organizing such a wonderful event for us. We were among the lucky few to have it’, and: ‘We should open a humanities site of our college where we can post videos of our role plays’.

## Discussion

Role plays as mentioned previously have been used for a variety of purposes in medical education. In the United Kingdom (UK), a mixed team of nursing and medical students were involved in an inter-professional pilot learning project on breaking bad news using role plays ^24^. Sufficient trust for learning between medical and nursing students ensued despite the briefness of the program. Our MH module was restricted to only medical students. Last year the college also admitted undergraduate dental students who had however joined the course late. An inter-professional MH module can be considered from the next academic session.

The high enjoyment scores are a matter of satisfaction. MH was widely regarded by this group as an enjoyable activity which provides a zone of comfort and relaxation, which further supports previous findings ^25^. We feel this aspect of MH is important to maintain student interest and participation and we are therefore satisfied with their responses regarding the enjoyment of the sessions. The MH module is not a formal part of our curriculum and is not assessed. However, the module remains popular with students; attendance at sessions is over 80% during the year the module was conducted and students feel it serves as a welcome break from their routine.

Role plays have also been used as a teaching strategy in Pakistan in community medicine ^26^. A number of benefits were noted by the participants. Role plays were mentioned as the most effective method of teaching; it improved their knowledge of the subject and said it would improve their clinical performance. Role plays would improve their communication skills, were regarded as a feasible method of andragogy and provoked critical thinking about the subject. Role plays have also been used in specific disciplines and amongst third and fourth year students. At MCOMS ^5^ and KISTMC ^6^ role plays were used in pharmacology to teach students to communicate with simulated patients and provide drug and non-drug information. In the present module, role plays were used to explore different issues and to familiarize students with the perspective of different characters involved. Students could experience first-hand what it meant to have a seriously ill child, the problems of the mentally ill, and the status of homosexuals in society among others. We debriefed the student participants occasionally and concentrated on how students felt playing different characters in the role plays. Improving communication skills of students was not a primary objective though it may have been one of the indirect benefits.

In the University of Chicago, a senior seminar is offered as a four week course in the fourth year to develop fluency in handling conflict and negotiation ^27^. This senior seminar also helps in understanding the elements of persuasive communication. In our module many of the case scenarios did address conflict and negotiation but no special training was provided to the students in these areas.

Students, despite a certain amount of reluctance, accepted that sexual and reproductive issues were important and should be addressed during the module. This was in contrast to the observation during a previous module conducted for faculty members. Faculty members were uncomfortable with sexual issues which they felt were ‘embarrassing’ ^28^. We are happy that first year students realized the important role of these issues in health and that keeping them ‘hidden’ may lead to more problems. The role plays also taught students to interact in a mature and cooperative manner with the opposite gender.

Training in simulated situations under the guidance and support of facilitators is becoming important in medicine. Role plays provide a safe and low risk learning environment for communication skills ^4^ and can serve to contribute life and a feeling of immediacy and involvement to academic situations. Role plays are rich in cognitive material, understanding and enacting the role play requires more information than what is provided, the problem unfolds and becomes richer over time, there is no single right way to tackle the problem, decisions have to be made in the absence of definitive knowledge and many solutions may exist ^29^. Debriefing participants and providing feedback on performance has been recommended ^4^. Due to time constraints we were not able to do debriefing for all role plays. Time continues to be a problem and we could consider increasing the duration to two hours in future. However, this will reduce time available for early clinical exposure. Another problem faced was that not many faculty members were interested in acting as facilitators. MH is still regarded as something ‘extra’ and not falling within strict subject boundaries. It may also be regarded as extra work which is not rewarded.

The study had limitations. Feedback was obtained using a semi-structured questionnaire. The data obtained was not triangulated with other sources. The questionnaire was not validated. Certain respondents may have had difficulty in understanding specific questions though facilitators were present to provide support. Participants could have had difficulties recalling role plays performed over a six month period.

## Conclusion

The study provides valuable feedback about the use of role plays in MH in a medical school in a developing country. Student feedback was positive and they wanted role plays to be used in future modules. Problems in small groups were resolved democratically and role plays introduced students to social and health issues in Nepal and prepared them for future practice. Role plays improved communication skills and explored sexual and reproductive issues. Role plays are not resource intensive and can be easily conducted in medical schools in developing nations with existing resources. Role plays can also be used in communication skills training, breaking bad news, clinical pharmacology and addressing community health issues among others. Role plays have an important role in educating future doctors and should be more widely used in medical schools especially in South Asia and other developing countries.

## References

[1] Hooker C: The medical humanities a brief introduction. *Aust Fam Physician.* 2008;37(5):369–370 18464968

[2] Shankar PR: A Voluntary Medical Humanities Module in a Medical College in Western Nepal: Participant feedback. *Teach Learn Med.* 2009;21(3):248–253 10.1080/10401330903020605 20183346

[3] Shankar R Piryani RM: Three years of Medical Humanities at a Nepalese medical school. *Educ Health.* 2011;24(1):535 21710422

[4] Joyner B Young L: Teaching medical students using role play: twelve tips for successful role plays. *Med Teach.* 2006;28(3):225–229 10.1080/01421590600711252 16753719

[5] Shankar PR Dubey AK Mishra M: Student attitudes towards communication skills learning in a medical college in western Nepal. *Educ Health.* 2006;19(1):71–84 16531304 10.1080/13576280500534693

[6] Shankar PR Jha N Bajracharya O: Teaching Pharmacology at a Nepalese Medical School: The Student Perspective. *Australas Med J.* 2010;1:14–22

[7] Shankar PR: Using case scenarios and role plays to explore issues of human sexuality. *Educ Health.* 2008;21(3):108 19967635

[8] Gupta S Singh S: Confluence: understanding medical humanities through street theatre. *Med Humanit.* 2011;37(2):127–8 10.1136/jmh.2010.006973 21778289

[9] Fertleman C Gibbs J Eisen S: Video improved role play for teaching communication skills. *Med Educ.* 2005;39(11):1155–1156 10.1111/j.1365-2929.2005.02283.x 16262828

[10] Van Ments M: The Effective Use of role play.London: Kogan Page1999 Reference Source

[11] Rosenbaum ME Ferguson KJ Lobas JG: Teaching medical students and residents skills for delivering bad news: a review of strategies. *Acad Med.* 2004;79(2):107–117 14744709 10.1097/00001888-200402000-00002

[12] Kopecky-Wenzel M Maier EM Muntau AC: Breaking bad news--a video-based training unit for medical students. *Z Kinder Jugendpsychiatr Psychother.* 2009;37(2):139–44 10.1024/1422-4917.37.2.139 19402001

[13] Nikendei C Kraus B Schrauth M: Integration of role playing into technical skills training: a randomized controlled trial. *Med Teach.* 2007;29(9):956–960 10.1080/01421590701601543 18158671

[14] Sherina HN Chia YC: Communication skills teaching in primary care medicine. *Med J Malaysia.* 2002;57(Suppl E):74–77 12733197

[15] Ng CJ McCarthy SA: Teaching medical students how to take a sexual history and discuss sexual health issues. *Med J Malaysia.* 2002;57(Suppl E):44–51 12733193

[16] Shankar PR Dubey AK Subish P: Critical evaluation of drug promotion using role plays. *Med Educ.* 2006;40(5):472 10.1111/j.1365-2929.2006.02429.x 16635142

[17] Downie RS: Literature and Medicine. *J Med Ethics.* 1991;17(2):93–6, 98. 10.1136/jme.17.2.93 1870090 PMC1376005

[18] Karki A Courneya CA Woollard R: Training of physicians for improving rural health care in Nepal: Building bridges to address the urban-rural gap. 2009

[19] Shankar PR Piryani RM Upadhyay-Dhungel K: Student feedback on the use of paintings in Sparshanam, the Medical Humanities module at KIST Medical College, Nepal. *BMC Med Educ.* 2011;11:9 10.1186/1472-6920-11-9 21385427 PMC3061959

[20] Huda S: Sex trafficking in South Asia. *Int J Gynecol Obstet.* 2006;94(3):374–81 10.1016/j.ijgo.2006.04.027 16846602

[21] Shapiro J Rucker L Robitshek D: Teaching the art of doctoring: an innovative medical student elective. *Med Teach.* 2006;28(1):30–35 10.1080/01421590600568348 16627319

[22] Wofford JL Ohl CA: Teaching appropriate interactions with pharmaceutical company representatives: the impact of an innovative workshop on student attitudes. *BMC Med Educ.* 2005;5(1):5 10.1186/1472-6920-5-5 15698480 PMC549188

[23] Shankar PR Singh KK Dhakal A: Transcripts of a medical education in humanities module: selection of role plays. *International Journal of User Driven Healthcare.* 2012;2(3):63–76 Reference Source

[24] Holsbrink-Engels GA: Using a computer learning environment for initial training in dealing with social-communicative problems. *Br J Educ Technol.* 2001;32(1):53–67 Reference Source

[25] Shankar PR: Medical HumanitiesIn R Biswas. & CM Martin (Eds.) User-driven healthcare and narrative medicine: utilizing collaborative social networks and technologies. (pp. 210–227). Hershey, PA: Medical Information Science Reference2011 10.4018/978-1-60960-097-6.ch016

[26] Manzoor I Mukhtar F Hashmi NR: Medical students’ perspective about role plays as a teaching strategy in community medicine. *J Coll Physicians Surg Pak.* 2012;22(4):222–5 22482377

[27] Ang M: Advanced communication skills: conflict management and persuasion. *Acad Med.* 2002;77(11):1166 12431946 10.1097/00001888-200211000-00034

[28] Shankar PR: Design the shoe according to the foot! *Clin Teach.* 2009;6(2):67–8 10.1111/j.1743-498X.2009.00269.x

[29] Wakefield A Cocksedge S Boggis C: Breaking bad news: qualitative evaluation of an interprofessional learning opportunity. *Med Teach.* 2006;28(1):53–58 10.1080/01421590500312805 16627325

