# Supplementary material for: Student feedback about the use of role plays in Sparshanam, a medical humanities module
Source: F1000Res. 2012 Dec 13;1:65. [Version 1] doi: 10.12688/f1000research.1-65.v1 (PMC3752645; doi:10.12688/f1000research.1-65.v1)
Supplement: Questionnaire student feedback about the use of role plays — Questionnaire used to determine student enjoyment and usefulness scores of role plays used during the medical humanities at KIST Medical College, Nepal [file f1000research-1-237-s0000.tgz › Questionnaire_student_feedback_about_the_use_of_role_plays.pdf]

**Student feedback about the use of role-plays in Sparshanam, the KISTMC Medical Humanities module**

Gender: M/F Self-financing/Scholarship

Give TWO overall comments about the use of role-plays in Sparshanam:

Have you exposed to the use of role-plays for educational objectives before? If yes, give details.

According to you how did role-plays help in realizing the objectives of the module? (Two points)

Are you aware of the use of role-plays in medical education elsewhere? If yes, give mention them.

Did you enjoy the use of role-plays in the module? Grade on a scale of 1 to 5 with 1 being least and 5 being most enjoyable.

Do you feel the scenarios covered in the role-plays were appropriate?

Give your reasons.

Give TWO suggestions to make use of role-plays more useful.

Which of the various role-plays used could you identify with the most? Why?

Which of the various role-plays used could you identify with the least? Why?

Did you have any difficulties regarding planning and enacting the role-plays?

If yes, then how did you overcome them?

Do you feel sexual and reproductive issues should be addressed using role-plays?

Do these issues create problems in enacting the role-plays? If yes, how did you overcome these problems?

Do you feel role-plays should be used in future modules?

Mention TWO advantages of role-plays for medical students.

How would you rate the usefulness of role-plays in the module on a scale of 1 to 5 (with 1 being least and 5 being most useful)

Give TWO suggestions to further improve the use of role-plays in future

**Any other comments:**

**Thank you for completing the questionnaire. It is very much appreciated!**
