# Supplementary material for: Student feedback about the use of role plays in Sparshanam, a medical humanities module
Source: F1000Res. 2012 Dec 13;1:65. [Version 1] doi: 10.12688/f1000research.1-65.v1 (PMC3752645; doi:10.12688/f1000research.1-65.v1)
Supplement: Peer review history

# Peer review: Referee response for version 1

**Published:** 2013-01-09

**Authors:** Ramalingam Sankran

**Affiliations:** PSG Institute of Medical Sciences and Research, Coimbatore, India, India

I have read this submission and think that it is a good piece of work done in Nepal. It is kind of pioneering medical education in South Asia.

I have read this submission. I believe that I have an appropriate level of expertise to confirm that it is of an acceptable scientific standard.

# Peer review: Referee response for version 1

**Published:** 2012-12-14

**Authors:** Supten Sarbadhikari

**Affiliations:** Supten Institute, Tamil Nadu, India

This is a commendable work on evaluating the student feedback for role plays to identify and interpret the learning objectives for empathy, what it means to be sick in Nepal, the doctor, the patient and the doctor-patient relationship.

The authors make a strong case towards the use of role plays for educating future doctors and rightly recommend that they should be more widely used in medical schools especially in South Asia and other developing countries.

I have read this submission. I believe that I have an appropriate level of expertise to confirm that it is of an acceptable scientific standard.
